# Supplementary material for: The interactome of intact mitochondria by cross-linking mass spectrometry provides evidence for coexisting respiratory supercomplexes
Source: Mol Cell Proteomics. 2017 Dec 8;17(2):216–32. doi: 10.1074/mcp.RA117.000470 (PMC5795388; doi:10.1074/mcp.RA117.000470)
Supplement: Supplemental Data [file supp_RA117.000470_133922_0_supp_23978_fzffwf.zip › spectra_annotation/mito_spectra_annotation/493-1-3-1-6-1.pdf]

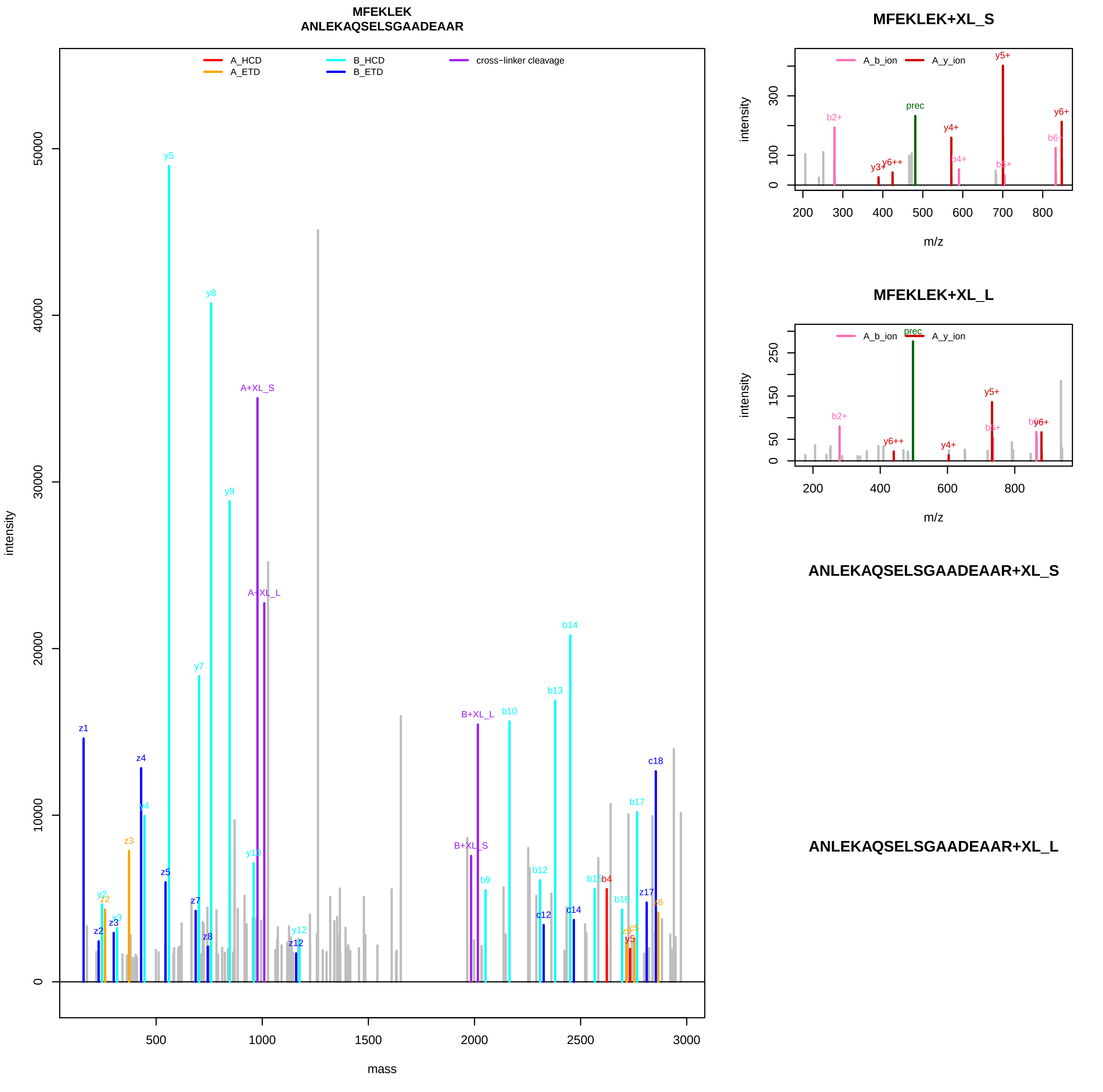
MFEKLEK
ANLEKAQSELSGAADEAAR
A_HCD
A_ETD
B_HCD
B_ETD
cross−linker cleavage
intensity
mass
0
10000
20000
30000
40000
50000
500
1000
1500
2000
2500
3000
z1
z2
y2
z2
z3
y3
z3
z4
y4
z5
y5
y7
z7
z8
y8
y9
y10
A+XL_S
A+XL_L
z12
y12
B+XL_S
B+XL_L
b9
b10
c12
b12
b13
c14
b14
b15
b4
b16
y5
c5
z5
b17
z17
c18
z6
MFEKLEK+XL_S
A_b_ion
A_y_ion
intensity
m/z
b2+
y3+
y6++
prec
y4+
b4+
y5+
b5+
b6+
y6+
MFEKLEK+XL_L
A_b_ion
A_y_ion
intensity
m/z
b2+
y6++
prec
y4+
y5+
b5+
b6+
y6+
ANLEKAQSELSGAADEAAR+XL_S
ANLEKAQSELSGAADEAAR+XL_L
